# Obesity and Mental Health: A Longitudinal, Cross-Cultural Examination in Germany and China

**DOI:** 10.3389/fpsyg.2021.712567

**Published:** 2021-09-21

**Authors:** Kristen L. Lavallee, Xiao Chi Zhang, Silvia Schneider, Jürgen Margraf

**Affiliations:** Mental Health Research and Treatment Center, Ruhr-Universität Bochum, Bochum, Germany

**Keywords:** positive mental health, depression, anxiety, longitudinal, cross-cultural, negative mental health

## Abstract

The present study examines the relationship between obesity and mental health using longitudinal data. Participants with data at baseline and one-year follow-up were included from two countries: Germany (364) and China (9007). A series of structural equation models with three mediators and one moderator were conducted separately for female and male students in Germany and China. Zero-order correlations indicated that overweight/obesity was significantly related to later depression and anxiety in Chinese males. Additional effects of obesity on later mental health flowed through effects on attractiveness (Chinese and German females, and Chinese males), physical health (Chinese males), and life satisfaction (German females). Though overweight/obesity is related to mental health across many other studies, results in this study yield total effects between overweight/obesity and follow-up mental health only in Chinese males. The relationship between overweight/obesity and follow-up mental health was significantly mediated by follow-up attractiveness, or health state, or life satisfaction in German females, Chinese females, and Chinese male students, with no significant indirect effects found in German male students. This highlights the possible importance of culture in examining these effects.

## Obesity and Mental Health: A Longitudinal, Cross-Cultural Examination

A preponderance of high-calorie, low-nutrient, and processed food, combined with increasingly sedentary lifestyles, has catapulted obesity rates to all-time highs. Pediatric rates for the newest additions to the population emerging from toddlerhood, young children between ages 5–9, are now at 10.4% in France, 15.1% in Brazil, 17.5% in China and Mexico, 22.7% in the US, and as high as 36.3% in the Pacific Islands (NCD Risk Factor Collaboration, 2017), affecting approximately one in five young people globally (Baker et al., 2017). Recent years have seen an important cultural shift away from weight and size to focusing on health. This shift toward health and away from a focus on size has been effective at improving overall health outcomes, reducing stigma, and maintaining self-esteem (Bacon, 2010). Nevertheless, overall rates of obesity have continued to rise and the consequences of obesity on physical and mental health remain. Many physical ailments and conditions, including obesity, are associated with serious mental disorders (Iacovides and Siamouli, 2008), and in particular anxiety and depression. In people with obesity, feelings of shame, guilt, and social anxiety are common and can be particularly prevalent in those with Binge Eating Disorder, as they are directly related to the disorder (Albohn-Kuhne and Rief, 2011). However, obesity is also more common in those with mental health concerns not typically considered part of disordered eating, such as schizophrenia (Bradshaw and Mairs, 2014), and depression (Chauvet-Gelinier et al., 2019). Some weight gain is associated with medications to treat mental disorders (Daumit et al., 2003; Bradshaw and Mairs, 2014), but medications do not explain all of the effects (Allison et al., 2009; Holt and Peveler, 2009). Further, the relationship between obesity and mental disorder has been investigated primarily in Western populations, leaving cross-cultural differences underexplored. The relationship between obesity and positive mental health, or protective factors, if any, remains largely uncharted.

### Obesity and Mental Disorder

Anxiety and obesity are the two most common health complaints and are often co-occurring. Overweight is associated with increased anxiety and depression with moderate effects (OR in the range of 1.2–1.5) across 13 different countries, concentrated in those with more severe obesity and in females in a large-scale survey within the World Mental Health Surveys (Scott et al., 2008). Across 25 studies included in one large meta-analysis, the frequency of anxiety occurred at an odds ratio of 1.30 in those with obesity and 1.10 in those with overweight. Aggregated effects were similar across men and women in this meta-analysis (Amiri and Behnezhad, 2019). A slightly older meta-analysis found similar effect sizes across 16 studies (2 prospective, with the remaining 14 cross-sectional), with an odds ratio of 1.4 for the association between obesity and anxiety (Gariepy et al., 2010). Obesity is also related to mood disorders. In a community study of over 36,000 Canadians, lifetime major depression was related to concurrent obesity with an odds ratio of 1.46 in females, though not related to obesity in males in this study (McIntyre et al., 2006).

Longitudinal research indicates a bidirectional relationship between obesity and mental health. One 11-year longitudinal study of the effects of mental health on weight gain and obesity in over 25,000 Norwegian adults found that the presence of anxiety or depression at baseline predicted increased weight gain and increased incidence of initial obesity over time (Brumpton et al., 2013). A comprehensive meta-analysis of the longitudinal bidirectional relationship between obesity/overweight and depression across 15 studies demonstrated that depression was predicted by both baseline obesity (odds ratio 1.55; stronger for Americans than Europeans) and baseline overweight (odds ratio 1.27). Further, baseline depression predicted later obesity (odds ratio 1.58), though not overweight. Moderators of these effects across studies in the meta-analysis, such as sex, age, and depression severity, were not significant, indicating that the link between depression and obesity was present regardless of sex, age, and severity of depression. The effects were significant for adults, but not teens in this meta-analysis (Luppino et al., 2010). Another more recent review found obesity linked to both mental distress and sexual dysfunction but found that causal links were difficult to draw from the existing research in the area of sexual dysfunction (Esfahani and Pal, 2018). While the Luppino meta-analysis did not find gender differences (Luppino et al., 2010), some individual studies have. Anxiety was a particular risk factor for later obesity in adolescent males in one study of youth aged 11–17 in the United States (Roberts and Duong, 2016). However, another study found significant longitudinal effects of obesity on increased risk for subsequent depression (adjusted hazard ratio, *HR*=3.9) and anxiety disorder (*HR*=3.8) for United States female adolescents, but not for males (Anderson et al., 2007). It may be that gender effects, while sometimes present in individual studies, average out to no effect when taken together across studies, or may become evident only in individual studies illuminating effects for teens, which Luppino did not find when examining effects across studies (Luppino et al., 2010).

Though the Luppino meta-analysis did not find effects for teens (Luppino et al., 2010), other research does point to some association between obesity and mental health in children and teens. For example, a large-scale population study of over 43,000 United States children found a depression rate of 15% for overweight, and 16% for obesity, with those classified as obese reporting increased internalizing (AOR 1.59) and externalizing (AOR 1.33) problems (Halfon et al., 2013). In a study of teens with severe mental illness, the teens were 30% likely to have obesity, and 55.5% likely to have overweight or obesity, rates approximately double national United States averages. Greater obesity in this study was related to lack of private health insurance (a stand-in for poverty), smoking, and antidepressant and antipsychotic medication use (Gracious et al., 2010). In another study of mental health in overweight and obese teens, the relationship between obesity and mental distress increased with severity of obesity (Calderon et al., 2009).

### Mechanisms Facilitating the Relationship Between Obesity and Mental Disorder

The relationship between obesity and mental disorder, like most relationships, is multiply determined. A study comparing psychiatric outpatients to the general population found that, as expected, psychiatric outpatients are more likely to have obesity than those in the general population. However, obesity was not related to the presence of psychiatric illness in this study, but rather to other co-occurring demographic factors, such as older age, gender, and comorbid chronic illnesses, suggesting that part of the link between mental health and obesity can be related more to mediational or common factors than to obesity itself (Dickerson et al., 2006; de Las Cuevas et al., 2011). Social and lifestyle factors also play a role. A cross-sectional study found that the link between depression and obesity was partially mediated by lower physical and social activity in those with depression (de Wit et al., 2010). People with obesity are also highly stigmatized and the experience of social stigma can be related to increased depression in those with obesity (Myers and Rosen, 1999).

Medical studies and studies of animals indicate that effects of obesity on mental health may not be purely social but have a strong biological component. Obesity-linked, hypercaloric (in particular high-sugar, high refined fat) diets appear to create vulnerability to the development of mental disorder, such as anxiety, *via* their effects on neuroendocrine stress and the neural circuitry that supports the regulation of emotions (Baker et al., 2017; de Noronha et al., 2017). Obesity-prone rats (especially males) experience elevated anxiety upon gaining weight, suggesting a biological relationship between obesity and anxiety that extends beyond the social challenges that cloud the ability to determine direction of effects in humans (Alonso-Caraballo et al., 2019). In another study of mice fed a standard diet vs. a Western diet (defined as palatable, energy-dense food) for 20weeks, mice fed a Western diet displayed impairments in spatial recognition memory and increased anxious behavior, as well as more susceptibility to depressive behavior after being immune suppressed (Andre et al., 2014). Mouse studies indicate that even exposure of a fetus to diet-induced maternal obesity can produce increased anxiety and impaired stress coping in offspring 3months (young) and 12months (aged) after birth, due to impairments in glucocorticoid receptors in the hypothalamus (Balsevich et al., 2016). The anxiety that comes with obesity appears to be reducible in animal models *via* measures to reduce chronic inflammation, such as dietary restriction and ibuprofen (Fourrier et al., 2019). Similarly, in humans, chronic inflammation, in the form of increased cytokines, such as tumor necrosis factor α and interleukin (IL)-1, often linked to diet and obesity, is associated with increased depression (Berk et al., 2013). Gastric bypass surgery for morbid obesity not only reduces obesity, but also improves mental health as well (Frezza et al., 2007). Further, decreasing Obesity Hypoventilation Syndrome in those with obesity *via* positive airway pressure therapy reduces symptoms of anxiety and depression (Argun Baris et al., 2016), again pointing to a reduction in obesity and obesity-related symptoms being related to a reduction in mental distress.

### Cross-Cultural Differences

While meta-analyses have shown some fairly consistent results, much of the research has been conducted with Western North American and European populations. However, there appear to be some important, and underexplored cultural differences in the appearance of these effects in different countries and cultures. A comparison of Caucasians, Latinos, and African Americans indicates that later obesity is predicted similarly across groups by earlier anxiety and mood disorders, but that strength and significance are slightly stronger for Caucasians than for Latinos and African Americans (Bodenlos et al., 2011). In a study of children with obesity in Brazil, where there is a high prevalence of pediatric obesity (Aiello et al., 2015), children with obesity were not found to be more anxious than children in other weight categories (Araujo et al., 2017). Interestingly, there appear to be some class differences in the effects of obesity on mental health in certain countries, but not others. Obesity is concurrently linked to higher rates of several mental disorders in German women, including anxiety, regardless of socioeconomic status (Becker et al., 2001). However, a study in Mexico, a country with some of the highest rates of obesity in the world, found that while obesity was linked to depression in more affluent people, it was positively related to mental health in the poor. It was hypothesized that obesity is likely to be more accepted and less stigmatized among the poor in certain countries (Bargain and Zeidan, 2019), perhaps those where resources are seen as more scarce, or where obesity is more common. Finally, in a study of Chinese children, being an only child (which remains more common in China due to the now-expired one-child policy) is related to greater obesity. However, while obesity is related to more depression in sibling pairs, only children (i.e., singletons) with obesity reported lower depression than children with obesity in sibling pairs. Children without overweight did not differ in depression according to sibling relationship. There were no differences in anxiety among children with or without obesity or sibling pairs in this study (Peng et al., 2019). In sum, cultural differences exist in the relationship between obesity and mental health, with the relationship potentially reversed under certain socioeconomic and structural conditions.

### Obesity and Positive Mental Health Facets

While historically, mental health has been defined *de facto* as the absence of psychopathology (Keyes, 2005; Prisciandaro and Roberts, 2009; Wittchen et al., 2011), this view is now recognized increasingly as overly simplistic, with positive mental health being a distinct and important construct for understanding the human condition (Prisciandaro and Roberts, 2009; Wittchen et al., 2011). A very rare few studies have found the potential for positive mental health or lower mental disorder accompanying obesity under certain circumstances, such as in the case of the poor in Mexico (Bargain and Zeidan, 2019), singletons in China (Peng et al., 2019), or no effect as in the case of the children in Brazil (Araujo et al., 2017). However, no known study has examined the relationship between obesity and positive aspects of mental health, such as sense of wellbeing, life satisfaction, or happiness. Traits, such as resilience and personal values, as well as states, such as current happiness and life satisfaction (Michael et al., 2007; Jacobi et al., 2014; Maercker et al., 2015; Lukat et al., 2016), are positively related to positive mental health (Haddadi and Besharat, 2010) and negatively associated with depressive symptoms (Brunwasser et al., 2009). It could be that an impact of obesity on resilience or happiness could mediate the relationship between obesity and mental distress or that the presence of positive traits or states could buffer those with obesity from mental distress. Further highlighting the importance of examining positive aspects of mental health separately from negative aspects, one recent large-scale longitudinal study of broad based mental disorders found that pathogenic predictors predict incidence and relapse of mental disorder diagnoses, while salutogenic factors predict remission over time (Lukat et al., 2016). It could be that obesity and positive mental health are related independently from mental distress and that the presence of salutogenic factors may serve as important buffers for those with overweight and obesity.

### Present Study

The present study is conducted within the “Bochum Optimism and Mental Health (BOOM) Project” (Margraf and Schneider, 2017), which aim to enhance integrated knowledge of the causes and consequences of positive mental health and mental health problems cross-culturally and over time. The present analysis, specifically, is a large-scale, cross-cultural, bi-national, and longitudinal investigation into the relationship between obesity and positive (life satisfaction, happiness, and resilience) and negative (anxiety and depression) mental health. Dimensional measures of mental health are examined, rather than categorical measures. Factors are examined across two time points (baseline and one-year follow-up), and across two countries (Germany and China). We limited the number of predictors for methodological reasons (Peduzzi et al., 1996). We hypothesized that obesity would generally be positively related to anxiety and depression, and negatively related to happiness over time. We expected health state, self-evaluation of attractiveness, and life satisfaction to partially but not fully mediate the links between obesity and mental state. We also explored resilience as a moderator of the pathways, and predicted a possible stronger effect of the moderator in China than in Germany, based on limited past research showing that the relationship between mental health and obesity can differ depending on family structure in China (Peng et al., 2019).

## Materials and Methods

### Procedure

The present study utilizes a subset of data from the BOOM project, a large-scale, cross-cultural, and longitudinal investigation of risk and protective factors in mental health (Maercker et al., 2015; Margraf and Schneider, 2017; Margraf et al., 2020). For a comprehensive overview of the full study design, aims, measures, and participants, see Margraf and Schneider (2017). The Ethics Committee of the Faculty of Psychology of the Ruhr-University Bochum approved the study. Approval to administer the questionnaires was granted by the Faculty of Psychology at Ruhr-University Bochum on May 12, 2011 and renewed on September 2013. The approvals for the German site were communicated to the participating Chinese Universities who acknowledged and accepted these approvals.

### Participants

#### Germany

The German sample consisted of 364 (1,040 had data at baseline and 395 of them had data at follow-up) student participants with BMI at baseline greater than 18.5 recruited from Ruhr-University Bochum in 2013. Students were assessed *via* online survey. German students were recruited *via* an e-mailed invitation with a link leading to an online questionnaire. The link was sent to all students enrolled at Ruhr-University Bochum. Participants were offered an incentive to take part in a draw for a gift coupon or a tablet computer. Participants had the opportunity at the end of the surveys contact the respective coordinators of the surveys with any questions or concerns. No participants contacted the researchers with concerns about the topic of obesity or the connection between obesity and mental health or disorder.

#### China

As the data were anonymized from the very beginning of data collection, no statement by an institutional board/ethics committee was required to collect data in China. The final Chinese sample consisted of 9,007 (12,057 had data at both time points, but those who with no BMI-value or a BMI-value under 18.5 were excluded, leaving a total of 9,007) university students with BMI at baseline greater than 18.5 from the Capital Normal University Beijing, the Hebei United University, Shanghai Normal University, Guizhou Finance and Economics University, and Nanjing University. Participants, mainly freshmen, were recruited during their first study month *via* an invitation mail. The response rate was 94.5%. Data were gathered *via* an online or a paper-pencil questionnaire administered in a group testing session. Participants received 10 RenMinBi (approximately 1.3 Euros) upon returning the questionnaire. As in Germany, participants in China had the opportunity at the end of the surveys contact the respective coordinators of the surveys with any questions or concerns. No participants contacted the researchers with concerns about the topic of obesity or the connection between obesity and mental health or disorder.

### Measures

#### Overview

As far as possible, established brief standard instruments, such as the Depression Anxiety Stress Scales (e.g., DASS 21), were used to measure the constructs of interest. For all questionnaires used in the analysis, validated German versions exist. Chinese versions of the measures were developed when needed, by using the customary translation-back-translation method as recommended (Brislin, 1970). In cases of discrepancies, this procedure was repeated by the study team until complete agreement was achieved. Measures can be grouped according to the overall design of the research program. Table 1 provides an overview.

**Table 1:** Descriptive statistics of sociodemographic variables and measures.

	China (*n* =9,007)		Germany (*n* =364)	
	Female(*n*=5,182)	Male(*n*=3,825)	Female(*n*=230)	Male(*n*=134)
Partnership BL				
Yes	732 (14.15%)	580 (15.18%)	123(53.48%)	56 (41.79%)
No	4,441 (85.85%)	3,242 (84.82%)	107 (46.52%)	78 (58.21%)
Overweight and Obesity BL				
Yes	223 (4.30%)	460 (12.03%)	40 (17.39%)	25 (18.66%)
No(= normal weight)	4,959 (95.70%)	3,365 (87.97%)	190 (82.61%)	109 (81.34%)
	Mean(SD)	Mean(SD)	Mean(SD)	Mean(SD)
Age_BL	19.58 (1.59)	19.79 (1.87)	20.60 (2.87)	20.48 (2.50)
FASII_BL	2.91 (2.14)	2.40 (2.10)	3.91 (1.76)	3.99 (1.76)
Resilience_BL	58.44 (8.03)	59.14 (8.89)	58.67 (9.33)	60.14 (8.05)
Attractiveness_FU	70.56 (16.15)	71.59 (19.24)	58.17 (23.68)	62.12 (18.35)
Health state_FU	83.58 (13.09)	83.11 (14.37)	72.92 (24.17)	75.78 (21.26)
Satisfaction with life_FU	23.05 (6.45)	22.43 (6.94)	24.48 (6.01)	23.76 (6.72)
Subjective happiness_FU	21.50 (4.11)	20.94 (4.87)	19.15 (4.50)	19.46 (4.10)
Anxiety_FU	3.00 (3.24)	3.29 (3.94)	3.40 (3.86)	2.70 (2.94)
Depression_FU	2.25 (3.04)	2.99 (3.98)	4.60 (4.70)	4.22 (4.00)

BL, baseline; FU, follow-up.

### General Outcomes

#### Overweight and Obesity

BMI was calculated based on self-reported weight and height, that is, the weight in kilograms divided by the square of the height in meters (kg/m^2^). Following recommendations of the (WHO, 2000), BMI was classified into four categories: 1=underweight (BMI<18.5kg/m^2^), 2=normal weight (18.5≤BMI<25kg/m^2^), 3=overweight (25≤BMI<30kg/m^2^), and 4=obese (BMI≥30kg/m^2^). Participants with underweight were not included in this study. The last two categories were merged into one “Overweight and Obesity” and coded as 1, while normal weight was coded as 0.

#### Subjective Happiness

Global subjective happiness was assessed using the four-item Subjective Happiness Scale (Lyubomirsky and Lepper, 1999). Participants responded on a 7-point Likert scale. The wording of anchor points depended on the question. Item scores are combined into a sum score with higher scores indicating higher happiness. Cronbach’s alphas at baseline were 0.829 (Germany) and 0.776 (China).

#### Depression, Anxiety, and Stress

Anxiety and depression were assessed using the widely used Depression Anxiety Stress Scales (DASS-21; Henry and Crawford, 2005). This short form of the DASS-42 (Lovibond and Lovibond, 1995) assesses a broad range of psychological distress symptoms. It is composed of three 7-item subscales for depressive, anxiety, and stress symptoms over the past week. The subscales may serve as outcome measures and as screening and monitoring instruments (Ng et al., 2007; Bayram and Bilgel, 2008; Dahm et al., 2013). Items are rated on a 4-point Likert scale ranging from 0 (did not apply to me at all) to 3 (applied to me very much or most of the time). Responses can be averaged within subscale or across all three for a total item score. Psychometric properties are well established in both clinical and non-clinical samples (Henry and Crawford, 2005; Ng et al., 2007) and are comparable for the short and long versions (Lovibond and Lovibond, 1995; Antony et al., 1998). In addition, the present authors have unpublished data on file that show the scale to be appropriate for cross-cultural research, with measurement invariance across cultures. In the present study, Cronbach’s alphas at baseline for depression were 0.886 (Germany) and 0.776 (China). Alphas for anxiety were 0.774 (Germany) and 0.737 (China).

### Mediators and Moderators

#### Resilience

Psychosocial stress resilience was assessed with an 11-item short version of the Wagnild and Young Resilience Scale (RS-14; RS-11; Wagnild and Young, 1993). Participants responded to items, such as “I usually manage one way or another” on a scale ranging from 1 (“I disagree”) to 7 (“I agree”). The RS-11 demonstrated good reliability and convergent validity in a German sample (Schumacher et al., 2005). Cronbach’s alphas at baseline were 0.891 (Germany) and 0.793 (China).

#### Attractiveness

Attractiveness was assessed using a single item: “How good or bad do you think you look compared to other people’s looks?” Participants rated themselves on a scale ranging from 0 (“completely ugly looking”) to 100 (“completely beautiful looking”).

#### Quality of Health

Overall current quality of health was assessed using the EuroQol (EQ-VAS; EuroQol Group, 1990, 2013; Brooks, 1996). Participants rated current health status on a scale ranging from 0 (worst imaginable health) to 100 (best imaginable health). Validity of EQ-VAS is indicated by convergence with the five dimensional version of the EuroQol (EuroQol 5D), with WHO-5, and known clinical groups across several countries.

#### Satisfaction With Life

The satisfaction with life scale (Diener et al., 1985) consists of five items focusing on global life satisfaction. Ratings on a 7-point Likert scale ranging from 1 (“strongly disagree”) to 7 (“strongly agree”) indicate agreement with each item. Scores were averaged across items. Cronbach’s alphas at follow-up were 0.881 (Germany) and 0.892 (China).

### Sociodemographic Predictors

#### Basic Sociodemographic Predictors

Sex, age, and relational status were assessed *via* self-report.

#### Family Affluence

To ensure sufficient comparability across vastly different cultures, the Family Affluence Scale II (FAS II; Boyce et al., 2006) served as the main cross-cultural measure of socioeconomic circumstances. The FAS II is, a four-item measure of family wealth, developed in the WHO Health Behavior in School-aged Children Study. Questions include (either with 2 or 3 response alternatives): “Does your family own a car, van or truck?”, “Do you have your own bedroom for yourself?”, “During the past 12months, how many times did you travel away on holiday with your family?”, and “How many computers does your family own?”. The FAS II total score is calculated by summing up the responses to these items. Convergent validity is established *via* correlations with the Gross National Product across 35 countries (Boyce et al., 2006). Cronbach’s alphas at baseline were 0.315 (Germany) and 0.640 (China), consistent with past research (Schnohr et al., 2008), indicating that the FAS II has low internal consistency. But FAS II still has the potential to address many of the limitations of other proxy measures of socioeconomic status currently being used in research (Kehoe and O’Hare, 2010).

## Statistical Analyses

All analyses are calculated with SPSS 24 (Corp, 2016) and PROCESS V3.4 (Hayes, 2013). A series of models are conducted for each country and gender separately to specify the relations between variables in this study. The program PROCESS was used to conduct our mediator-moderator analyses, and PROCESS does not produce measures of fit for the entire model (Hayes et al., 2017). Therefore, we did not report the model fit indices. Obesity and overweight at baseline are used as a predictor of happiness, depression, and anxiety at follow-up, while using attractiveness, health, and satisfaction with life at follow-up as mediators and resilience at baseline as moderator. Age, FAS II, and having a partner at baseline are all controlled in the models. Tests of moderation within path models are conducted. The bootstrapping method with bias corrected confidence interval (MacKinnon et al., 2004) is estimated to test the significance of mediators. In this study, the 95% confidence interval of the indirect effects is calculated with 5,000 bootstrap resamplings (Preacher and Hayes, 2008). As having obesity/overweight at baseline is coded as a dichotomous variable, no simple slope analyses are conducted for any significant interaction paths.

Missing values were generally between 0.0 and 3.3%, depending on the measure. Participants with missing data were deleted pairwise from the analyses (meaning they were deleted from analyses in which those questionnaires were used). Internal consistency is computed with Cronbach’s α coefficient. Cronbach’s α>0.70 indicates acceptable, >0.80 good, and >0.90 excellent internal consistency (Kline, 2000).

## Results

### Descriptive Statistics

Table 1 presents data on participant demographics and descriptive statistics for the predictors and outcomes at baseline. Approximately 49% of Germans were in a partnership, while only 14.6% of Chinese participants were in a partnership. About 18% of Germans in the current study had overweight or obesity, while 7.6% of Chinese participants had the same. The German participants rated their family affluence as slightly higher, their attractiveness as lower, health state lower, and depression higher than the Chinese participants. Between country differences were not evaluated for significance. Chinese participants had a mean age of 19.67(*SD*=1.72), and German participants had a mean age of 20.55 (*SD*=2.74).

### Correlations

The correlations among the psychological predictors are shown in Table 2A (Germany) and Table 2B (China). Pearson’s zero-order correlations indicated that overweight/obesity was significantly related to lower family affluence attractiveness, health, and life satisfaction, but not depression or anxiety in German female participants. Overweight/obesity was only significantly related to higher family affluence in German male participants. It was related to slightly higher family affluence, lower resilience, and lower attractiveness, but not to life satisfaction, depression, or anxiety in Chinese females. Overweight/obesity was, however, related to higher family affluence, later depression, and anxiety in Chinese males.

**Table 2A:** Bivariate correlations among the psychological predictors within Germany, with female below diagonal and male above diagonal.

	Partnership BL	Overweight and Obesity BL	Age BL	FASII BL	Resilience BL	Attractiveness FU	Health state FU	Satisfaction with life FU	Subjective happiness FU	Anxiety FU	Depression FU
Partnership BL	1	−0.056	0.123	−0.044	−0.015	−0.038	−0.025	0.166	0.198^*^	−0.043	−0.058
OverweightandObesity BL	−0.124	1	0.169	0.178^*^	−0.035	−0.118	0.006	0.026	0.036	0.141	0.143
Age_BL	0.084	0.177^**^	1	−0.175^*^	−0.138	−0.12	−0.199^*^	−0.216^*^	−0.087	0.156	0.301^**^
FASII_BL	0.041	−0.140^*^	−0.151^*^	1	0.08	0.134	0.11	0.203^*^	0.146	−0.042	−0.107
Resilience_BL	0.118	−0.093	0.071	0.126	1	0.206^*^	0.255^**^	0.434^**^	0.411^**^	−0.365^**^	−0.394^**^
Attractiveness_FU	0.092	−0.195^**^	−0.109	0.043	0.163^*^	1	0.283^**^	0.152	0.289^**^	−0.142	−0.231^**^
Health state_FU	0.099	−0.142^*^	−0.114	0.139^*^	0.066	0.367^**^	1	0.342^**^	0.284^**^	−0.341^**^	−0.351^**^
Satisfaction with life_FU	0.163^*^	−0.219^**^	−0.082	0.286^**^	0.416^**^	0.393^**^	0.279^**^	1	0.522^**^	−0.282^**^	−0.386^**^
Subjective happiness_FU	0.173^**^	−0.11	0.043	0.131^**^	0.455^**^	0.373^**^	0.178^**^	0.699^**^	1	−0.294^**^	−0.489^**^
Anxiety_FU	−0.109	0.079	0	−0.208^*^	−0.138^*^	−0.147^*^	−0.198^**^	−0.392^**^	−0.381^**^	1	0.665^**^
Depression_FU	−0.148^*^	0.109	0.02	−0.174^**^	−0.272^**^	−0.350^**^	−0.259^**^	−0.624^**^	−0.555^**^	0.670^*^	1

* *p*<0.05, two tailed;

** *p*<0.01, two tailed. BL = baseline, FU = follow-up

**Table 2B:** Bivariate correlations among the psychological predictors within China, with female below diagonal and male above diagonal.

	Partnership BL	Overweight and Obesity BL	Age BL	FASII BL	Resilience BL	Attractiveness FU	Health state FU	Satisfaction with life FU	Subjective happiness FU	Anxiety FU	Depression FU
Partnership BL	1	0.003	0.157^**^	0.106^**^	0.016	0.056^**^	0.033^*^	0.070^**^	0.051^**^	−0.019	−0.014
OverweightandObesity BL	−0.037^**^	1	0.006	0.121^**^	−0.024	−0.087^**^	−0.061^**^	0.03	−0.011	0.058^**^	0.055^**^
Age_BL	0.195^**^	−0.021	1	−0.154^**^	0.037^*^	0.055^**^	0.069^**^	0.022	0.022	−0.025	−0.022
FASII_BL	0.055^**^	0.037^**^	−0.148^**^	1	0.046^**^	−0.051^**^	−0.094^**^	0.094^**^	0.018	0.009	0.012
Resilience_BL	0.034^*^	−0.034^*^	0.034^**^	0.070^**^	1	0.137^**^	0.139^**^	0.216^**^	0.252^**^	−0.192^**^	−0.204^**^
Attractiveness_FU	0.093^**^	−0.031^*^	0.052^**^	0.052^**^	0.163^**^	1	0.356^**^	0.166^**^	0.257^**^	−0.159^**^	−0.176^**^
Health state_FU	0.013	−0.019	0.006	−0.080^**^	0.154^**^	0.270^**^	1	0.232^**^	0.319^**^	−0.255^**^	−0.260^**^
Satisfaction with life_FU	0.086^**^	0.009	0.044^*^	0.140^**^	0.194^**^	0.215^**^	0.204^**^	1	0.542^**^	−0.227^**^	−0.237^**^
Subjective happiness_FU	0.064^**^	−0.006	0.048^**^	0.054^**^	0.262^**^	0.255^**^	0.322^**^	0.492^**^	1	−0.339^**^	−0.371^**^
Anxiety_FU	−0.027	0	−0.064^**^	−0.044^**^	−0.184^**^	−0.156^**^	−0.286^**^	−0.237^**^	−0.320^**^	1	0.881^**^
Depression_FU	−0.038^**^	0.008	−0.043^**^	−0.039^**^	−0.210^**^	−0.167^**^	−0.286^**^	−0.262^**^	−0.364^**^	0.789^**^	1

* *p*<0.05, two tailed;

** *p*<0.01, two tailed. BL = baseline, FU = follow-up.

### Structural Equation Model

**Figure 1:**
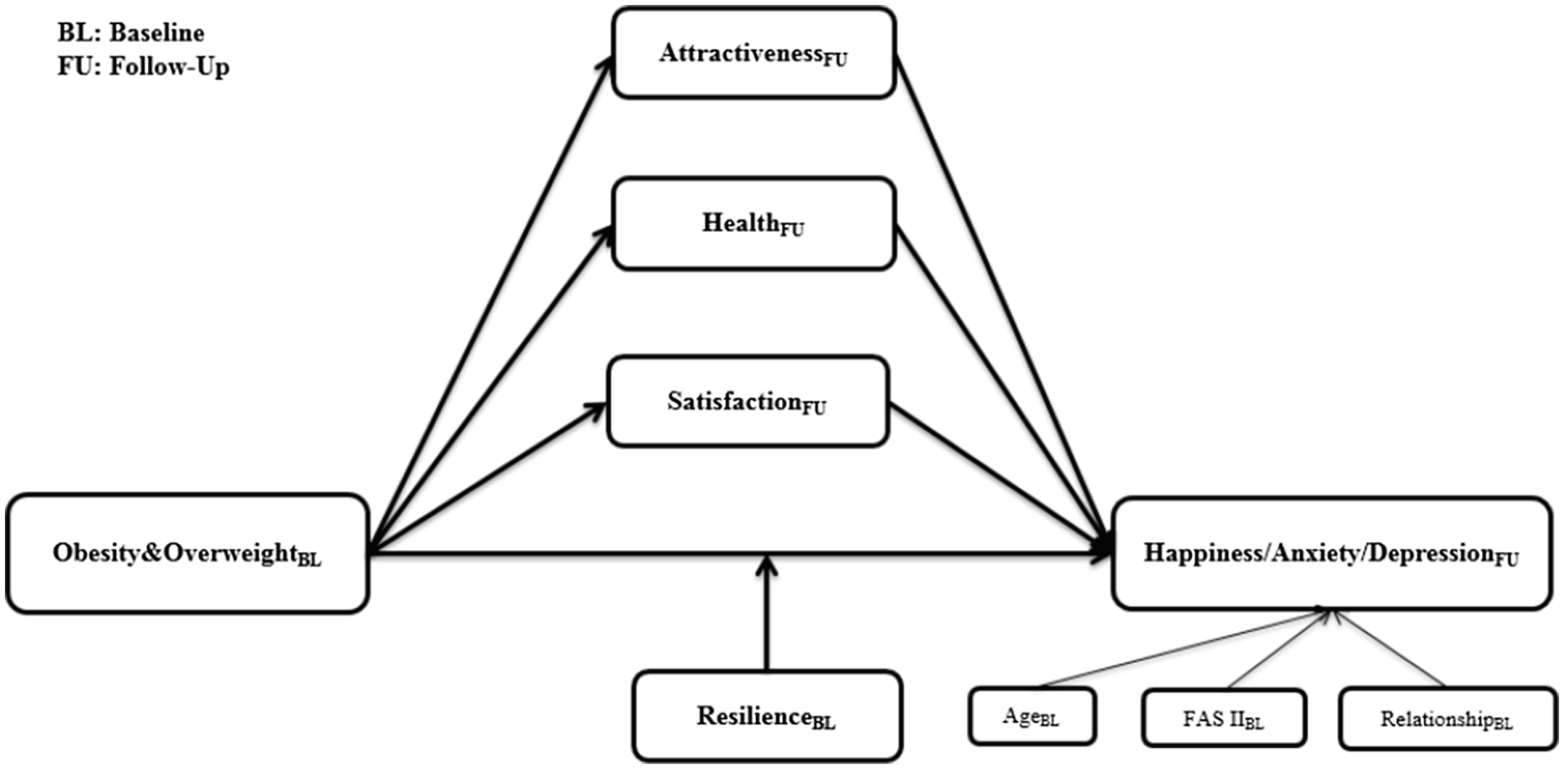
Model with attractiveness, health state, and satisfaction from follow-up as mediators and resilience from baseline as moderator.

The general model is presented in Figure 1. The model was tested for three different outcomes happiness, anxiety, and depression, and was tested separately for the German and Chinese samples. Table 3 indicates the effect strength and significance for each pathway with happiness as the outcome. Table 4 indicates the same model results, except with depression as the outcome. Table 5 indicates the model results with anxiety as the outcome.

**Table 3:** Summary of mediated and moderated regression analyses predicting happiness.

	China-Male		China-Female		Germany-Male		Germany-Female	
	B	SE	B	SE	B	SE	B	SE
OO_BL > Attractiveness_FU	−4.941^***^	1.025	−2.495^*^	1.145	−6.305	4.208	−11.538^**^	4.240
OO_BL > Health_FU	−2.262^**^	0.764	−0.455	0.932	1.386	4.871	−7.043	4.343
OO_BL > Satisfaction_FU	0.512	0.370	0.345	0.458	0.769	1.482	−2.588^*^	1.029
OO_BL > Happiness_FU	0.433	1.304	3.458^*^	1.647	−7.210	5.167	−3.722	3.719
Attractiveness_FU > Happiness_FU	0.025^***^	0.004	0.022^***^	0.003	0.038^*^	0.017	0.024^*^	0.010
Health_FU > Happiness_FU	0.055^***^	0.005	0.062^***^	0.004	0.012	0.015	−0.005	0.009
Satisfaction_FU > Happiness_FU	0.324^***^	0.010	0.250^***^	0.008	0.216^***^	0.052	0.450^***^	0.042
Resilience_BL > Happiness_FU	0.070^***^	0.008	0.076^***^	0.006	0.077	0.045	0.084^**^	0.026
Age_BL > Happiness_FU	−0.031	0.039	0.046	0.034	0.047	0.124	0.097	0.074
FASII_BL > Happiness_FU	−0.022	0.034	0.002	0.024	0.083	0.174	1.154	0.124
Relationship_BL > Happiness_FU	0.129	0.193	0.132	0.145	1.303^*^	0.606	0.419	0.422
*Moderation Effect*								
OO_BL^*^Resilience_BL	−0.007	0.022	−0.062^*^	0.028	0.130	0.085	0.075	0.064
*Indirect Effects*	Effect (SE)	Bootstrap-CI	Effect (SE)	Bootstrap-CI	Effect (SE)	Bootstrap-CI	Effect (SE)	Bootstrap-CI
Attractiveness_FU	**−0.124 (0.036)**	**(−0.201, −0.060)**	**−0.054 (0.025)**	**(−0.105, −0.009)**	−0.238 (0.219)	(−0.741, 0.091)	**−0.280 (0.194)**	**(−0.736, −0.006)**
Health_FU	**−0.123 (0.050)**	**(−0.226, −0.028)**	−0.028 (0.059)	(−0.145, 0.087)	0.016 (0.098)	(−0.187, 0.253)	0.034 (0.089)	(−0.165, 0.209)
Satisfaction_FU	0.166 (0.119)	(−0.072, 0.398)	0.086 (0.111)	(−0.132, 0.301)	0.166 (0.349)	(−0.417, 0.964)	**−1.164 (0.518)**	**(−2.176, −0.156)**

Significant effects in bold; B, unstandardized coefficient; and SE, standard error.

* *p*<0.05;

** *p*<0.01;

*** *p*<0.001. BL = baseline, FU = follow-up.

**Table 4:** Summary of mediated and moderated regression analyses predicting depression.

	China-Male		China-Female		Germany-Male		Germany-Female	
	B	SE	B	SE	B	SE	B	SE
OO_BL > Attractiveness_FU	−4.867^***^	1.031	−2.447^*^	1.149	−6.355	4.227	−11.883^**^	4.211
OO_BL > Health_FU	−2.251^**^	0.769	−0.435	0.939	1.327	4.908	−6.735	4.369
OO_BL-->Satisfaction_FU	0.518	0.373	0.326	0.459	0.710	1.482	−2.444^*^	1.027
OO_BL > Depression_FU	1.647	1.220	−0.740	1.343	1.249	5.395	−2.876	4.433
Attractiveness_FU > Depression_FU	−0.009^**^	0.004	−0.009^***^	0.003	−0.016	0.018	−0.019	0.012
Health_FU > Depression_FU	−0.052^***^	0.005	−0.050^***^	0.003	−0.034^*^	0.016	−0.015	0.011
Satisfaction_FU > Depression_FU	−0.094^***^	0.010	−0.083^***^	0.007	−0.097	0.054	−0.440^***^	0.051
Resilience_BL > Depression_FU	−0.057^***^	0.008	−0.048^***^	0.005	−0.113^*^	0.047	−0.016	0.031
Age_BL > Depression_FU	−0.020	0.036	−0.062^*^	0.027	0.277^*^	0.130	−0.062	0.089
FASII_BL > Depression_FU	0.014	0.031	−0.034	0.019	−0.037	0.181	0.025	0.150
Relationship_BL > Depression_FU	0.104	0.181	−0.079	0.118	−0.469	0.639	−0.306	0.506
*Moderation Effect*								
OO_BL^*^Resilience_BL	−0.021	0.021	0.015	0.023	−0.004	0.089	0.044	0.077
*Indirect effects*	Effect (SE)	Bootstrap-CI	Effect (SE)	Bootstrap-CI	Effect (SE)	Bootstrap-CI	Effect (SE)	Bootstrap-CI
Attractiveness_FU	**0.045 (0.025)**	**(0.003, 0.102)**	**0.022 (0.014)**	**(0.001, 0.053)**	0.102 (0.166)	(−0.127, 0.520)	0.230 (0.196)	(−0.068, 0.692)
Health_FU	**0.116 (0.047)**	**(0.030, 0.217)**	0.022 (0.048)	(−0.069, 0.120)	−0.045 (0.169)	(−0.400, 0.302)	0.100 (0.114)	(−0.040, 0.406)
Satisfaction_FU	−0.049 (0.034)	(−0.119, 0.018)	−0.027 (0.037)	(−0.010, 0.046)	−0.069 (0.183)	(−0.546, 0.200)	**1.074 (0.505)**	**(0.091, 2.055)**

Significant effects in bold; B, unstandardized coefficient; and SE, standard error.

* *p*<0.05;

** p<0.01;

*** *p*<0.001. BL = baseline, FU = follow-up.

**Table 5:** Summary of mediated and moderated regression analyses predicting anxiety.

	China-Male		China-Female		Germany-Male		Germany-Female	
	B	SE	B	SE	B	SE	B	SE
OO_BL > Attractiveness_FU	−4.730^***^	1.028	−2.548^*^	1.154	−6.305	4.208	−12.208^**^	4.185
OO_BL > Health_FU	−2.167^**^	0.765	−0.466	0.942	1.386	4.871	−7.734	4.360
OO_BL > Satisfaction_FU	0.506	0.372	−0.291	0.461	0.769	1.482	−2.484^*^	1.028
OO_BL-->Anxiety_FU	1.326	1.210	−0.770	1.473	5.832	4.141	−0.114	4.379
Attractiveness_FU > Anxiety_FU	−0.006	0.004	−0.010^***^	0.003	0.004	0.014	0.005	0.012
Health_FU > Anxiety_FU	−0.053^***^	0.005	−0.055^***^	0.003	−0.034^**^	0.012	−0.012	0.011
Satisfaction_FU > Anxiety_FU	−0.089^***^	0.010	−0.076^***^	0.007	−0.032	0.042	−0.236^***^	0.053
Resilience_BL > Anxiety_FU	−0.051^***^	0.008	−0.044^***^	0.006	−0.078^*^	0.036	0.012	0.030
Age_BL > Anxiety_FU	−0.025	0.036	−0.126^***^	0.030	0.048	0.100	−0.069	0.087
FASII_BL > Anxiety_FU	0.009	0.031	−0.055^**^	0.021	−0.008	0.139	−0.233	0.150
Relationship_BL > Anxiety_FU	0.062	0.179	0.080	0.128	−0.254	0.485	−0.119	0.510
*Moderation Effect*								
OO_BL^*^Resilience_BL	−0.014	0.020	0.011	0.025	−0.081	0.068	0.002	0.076
*Indirect Effects*	Effect (SE)	Bootstrap-CI	Effect (SE)	Bootstrap-CI	Effect (SE)	Bootstrap-CI	Effect (SE)	Bootstrap-CI
Attractiveness_FU	0.026 (0.022)	(−0.012, 0.072)	**0.025 (0.014)**	**(0.014, 0.003)**	−0.027 (0.103)	(−0.240, 0.193)	−0.059 (0.188)	(−0.471, 0.306)
Health_FU	**0.115 (0.049)**	**(0.025, 0.216)**	0.026 (0.054)	(−0.079, 0.134)	−0.046 (0.162)	(−0.345, 0.316)	0.092 (0.121)	(−0.062, 0.418)
Satisfaction_FU	−0.045 (0.033)	(−0.112, 0.017)	−0.022 (0.035)	(−0.089, 0.047)	−0.024 (0.092)	(−0.276, 0.099)	**0.587 (0.328)**	**(0.047, 1.324)**

Significant effects in bold; B, unstandardized coefficient; and SE, standard error.

* *p*<0.05;

** *p*<0.01;

*** *p*<0.001. BL = baseline, FU = follow-up.

Results indicated that in Chinese male students, attractiveness t2 and health state t2 are significant mediators for depression t2 and happiness t2. Further, health state t2 is a significant mediator for anxiety t2. In Chinese female students, attractiveness t2 is a significant mediator for depression t2, anxiety t2, and happiness t2. Further, resilience t1 is a significant moderator only for happiness t2. For Chinese females with low resilience at baseline, overweight/obesity is related to higher happiness at the follow-up than normal weight is. However, for Chinese females with high resilience at baseline, overweight/obesity leads to lower subjective happiness at the follow-up than normal weight. In German male students, there was no significant mediator or moderator. In German female students, satisfaction with life t2 is a significant mediator for depression t2 and anxiety t2. Further, attractiveness t2 and satisfaction with life t2 are significant mediators for happiness t2.

## Discussion

To our knowledge, this is the first longitudinal prospective study to examine the relationship between obesity and positive and mental health and obesity in a cross-national sample. Results from zero-order correlations indicated that overweight/obesity at baseline was related to lower concurrent family affluence and lower follow-up attractiveness, health, and life satisfaction, but not related to follow-up depression or anxiety in German female participants. Overweight/obesity was only significantly related to higher family affluence in German male participants. It was related to slightly higher family affluence, lower resilience, and lower attractiveness, but not to life satisfaction, depression, or anxiety in Chinese females. It was, however, related to increased family affluence, anxiety, and depression in Chinese males. The lack of associations between obesity and mental health in German men and women and Chinese women is in contrast to prior research. Indeed, several large-scale studies and meta-analyses have documented the relationship between overweight and increased anxiety and depression with moderate effects (OR in the range of 1.2–1.5) across 13 different countries in the World Mental Health Surveys (Scott et al., 2008), and between overweight and anxiety across 25 studies included in a large meta-analysis, with effects similar across sex (Amiri and Behnezhad, 2019), and across 16 studies in Gariepy et al. (2010). In a community study of over 36,000 Canadians, lifetime major depression was related to concurrent obesity with an odds ratio of 1.46 in females, though not related to obesity in males in this study (McIntyre et al., 2006). These findings make our own results even more perplexing, as we found a link between obesity and anxiety/depression in Chinese men, but not women. It could be that the sex difference result is dependent on culture, and we have no comprehensive study of Chinese sex differences until now. There are certainly cultural differences in these effects. A meta-analysis of the longitudinal bidirectional relationship between obesity/overweight and depression across 15 studies demonstrated that prediction of depression baseline obesity was stronger for Americans than Europeans. However, moderators of these effects across studies in the meta-analysis, including sex and age, were not significant (Luppino et al., 2010). Then again, some individual studies do find a sex effect, but the direction can be contradictory. Anxiety was a particular risk factor for later obesity in adolescent males in one study of United States teens (Roberts and Duong, 2016). Yet, another study found effects for United States female adolescents, but not for males (Anderson et al., 2007). Gender effects may be unstable, dependent on culture, or only apparent in teens, and perhaps college students, as we found in the present study.

Though zero-order correlations indicated that the expected obesity and mental health effects were only present in Chinese males, mediational analysis guidelines suggest that significant total effects (i.e., zero-order correlations) are not a necessary condition for examining mediational or indirect effects (Rucker et al., 2011). Thus, we did proceed to examine the relationships between obesity and outcomes in the planned structural equation models. The structural equation models indicated that in Chinese male students, attractiveness t2 and health state t2 were significant mediators for depression t2 and happiness t2. Further, health state t2 was a significant mediator for anxiety t2. That is, the relationship between obesity and mental health in Chinese males mediated by the time two assessments of one’s own attractiveness and health.

In Chinese female students, attractiveness t2 was a significant mediator for depression t2, anxiety t2, and happiness t2. That is, in Chinese females, the relationship between obesity and mental health was significantly mediated by later attractiveness. Further, resilience t1 is a significant moderator, but only for happiness t2. That is, for Chinese females with low resilience at baseline, overweight/obesity is related to higher happiness at the follow-up than normal weight is. However, for Chinese females with high resilience at baseline, overweight/obesity leads to lower subjective happiness at the follow-up than normal weight.

In German male students, there was no significant mediator or moderator. In German female students, satisfaction with life t2 is a significant mediator for depression t2 and anxiety t2. Further, attractiveness t2 and satisfaction with life t2 are significant mediators for happiness t2. That is, although there was not a direct link between obesity and mental health (anxiety and depression) in German females, the structural equation model pointed to some significant mediational effects flowing through life satisfaction (on depression, anxiety, and happiness) and attractiveness (on happiness). In sum, obesity was not related to later mental health in the zero-order correlations for any group except for Chinese males. In Chinese males, obesity was significantly correlated with later depression and anxiety. Obesity was related to later attractiveness (Chinese and German females, and Chinese males), health (German females and Chinese males), and life satisfaction (German females), which were, in turn, related to time two mental health. Further, for Chinese females with low resilience, obesity can correlate with higher later happiness, whereas for Chinese females with high resilience, obesity is related to lower later happiness.

While not all who are overweight experience mental distress, for those for whom obesity is related to mental distress, it may be the case that reductions in obesity could foster some improvements in mental health. Some research indicates that weight loss is associated with improvements in mental health functioning (Carmichael et al., 2001; Jarvholm et al., 2012), suggesting that a reduction in obesity status leads to a reduction in mental distress. However, other health scientists have found that a focus on weight reduction in interventions can be counter-productive in improving both physical and mental wellbeing and that a focus on improving health rather than reducing weight leads to better outcomes (Bacon, 2010).

The present study has a number of strengths, including the large sample size, its thorough assessments using standardized instruments and follow-up interview over a period of 17months. Because of the homogenous sample, age, gender, and socioeconomic characteristics were less likely to confound the effects of the psychological predictors. Moreover, we investigated the psychological predictors within a longitudinal design. Although there are several strengths associated with the study, there are also limitations. First, it is not possible to draw conclusions about causation, as this study did not employ an experimental design. Second, the present study examined effects in college students, and results may not be generalizable to populations of older or other non-college-attending adults. Third, obesity and overweight were coded as binary variables, when in reality weight exists on a linear scale. Fourth, the present study used self-report data. It may be that clinical reports of obesity and mental health would provide added reliability to the data. Fifth, there were fewer German than Chinese participants, and it may be that with more German participants, we would have had increased power to observe small effects. Sixth, this study used self-report measures, which may be subject to bias and are not as objective as multi-rater or observational methods. Seventh, participants were given an incentive to participate, which may have influenced the sample. Finally, while all ethical guidelines were followed, we agree with a limitation of the study pointed out by an anonymous reviewer: That is, participants filling out self-report questionnaires on mental health should be provided with a follow-up resource for mental health concerns.

Overweight/obesity is related to mental health across many studies and meta-analyses. However, in this study, total effects between overweight/obesity and follow-up mental health were only found significant in Chinese males. The effects between overweight/obesity and follow-up mental health were significantly mediated by follow-up attractiveness, or health state, or satisfaction in German females, Chinese females, and Chinese male students, but no significant indirect effects were found in German male students. This highlights the possible importance of culture in examining these effects. These results await replication and should be taken in the context of the broad literature on this topic. They may support newer foci on health and self-image rather than weight in treating mental health in obesity. The relationship between obesity and mental health in prior research still points to need for a multi-tiered public health approach to addressing mental health in those with obesity, including community intervention and prevention, as well as individual support (Bazyk and Winne, 2013).

## Data Availability Statement

The raw data supporting the conclusions of this article will be made available by the authors, without undue reservation.

## Ethics Statement

The studies involving human participants were reviewed and approved by the Ruhr-University Bochum. Written informed consent from the participants’ legal guardians/next of kin was not required to participate in this study in accordance with the national legislation and the institutional requirements.

## Author Contributions

KL wrote the first draft and revised the manuscript. XZ analyzed the data, designed the analyses, and wrote the results. SS and JM designed the study, managed the study, planned the analyses, and provided the funding. All authors contributed to the article and approved the submitted version.

## Funding

This study was supported by the Alexander von Humboldt Professorship awarded to Jürgen Margraf by the Alexander von Humboldt Foundation.

## Conflict of Interest

The authors declare that the research was conducted in the absence of any commercial or financial relationships that could be construed as a potential conflict of interest.

## Publisher’s Note

All claims expressed in this article are solely those of the authors and do not necessarily represent those of their affiliated organizations, or those of the publisher, the editors and the reviewers. Any product that may be evaluated in this article, or claim that may be made by its manufacturer, is not guaranteed or endorsed by the publisher.

## References

[ref1] Aiello A. M. Marques de Mello L. Souza Nunes M. Soares da Silva A. Nunes A. (2015). Prevalence of obesity in children and adolescents in Brazil: A meta-analysis of cross-sectional studies. Curr. Pediatr. Rev. 11, 36–42. doi: 10.2174/1573396311666150501003250, PMID: 25938377

[ref2] Albohn-Kuhne C. Rief W. (2011). Shame, guilt and social anxiety in obesity with binge-eating disorder. Psychother. Psychosom. Med. Psychol. 61, 412–417. doi: 10.1055/s-0031-1284334, PMID: 21800275

[ref3] Allison D. B. Newcomer J. W. Dunn A. L. Blumenthal J. A. Fabricatore A. N. Daumit G. L.. (2009). Obesity among those with mental disorders: a National Institute of Mental Health meeting report. Am. J. Prev. Med. 36, 341–350. doi: 10.1016/j.amepre.2008.11.020, PMID: 19285199

[ref4] Alonso-Caraballo Y. Hodgson K. J. Morgan S. A. Ferrario C. R. Vollbrecht P. J. (2019). Enhanced anxiety-like behavior emerges with weight gain in male and female obesity-susceptible rats. Behav. Brain Res. 360, 81–93. doi: 10.1016/j.bbr.2018.12.002, PMID: 30521928 PMC6462400

[ref5] Amiri S. Behnezhad S. (2019). Obesity and anxiety symptoms: a systematic review and meta-analysis. Neuropsychiatrie 33, 72–89. doi: 10.1007/s40211-019-0302-9, PMID: 30778841

[ref6] Anderson S. E. Cohen P. Naumova E. N. Jacques P. F. Must A. (2007). Adolescent obesity and risk for subsequent major depressive disorder and anxiety disorder: prospective evidence. Psychosom. Med. 69, 740–747. doi: 10.1097/PSY.0b013e31815580b4, PMID: 17942847

[ref7] Andre C. Dinel A. L. Ferreira G. Laye S. Castanon N. (2014). Diet-induced obesity progressively alters cognition, anxiety-like behavior and lipopolysaccharide-induced depressive-like behavior: focus on brain indoleamine 2,3-dioxygenase activation. Brain Behav. Immun. 41, 10–21. doi: 10.1016/j.bbi.2014.03.012 24681251

[ref8] Antony M. M. Bieling P. J. Cox B. J. Enns M. W. Swinson R. P. (1998). Psychometric properties of the 42-item and 21-item versions of the depression anxiety stress scales in clinical groups and a community sample. Psychol. Assess. 10, 176–181. doi: 10.1037/1040-3590.10.2.176

[ref9] Araujo D. S. Marquezin M. Barbosa T. S. Fonseca F. Fegadolli C. Castelo P. M. (2017). Assessment of quality of life, anxiety, socio-economic factors and caries experience in Brazilian children with overweight and obesity. Int. J. Dent. Hyg. 15, e156–e162. doi: 10.1111/idh.12248, PMID: 27699998

[ref10] Argun Baris S. Tuncel D. Ozerdem C. Kutlu H. Onyilmaz T. Basyigit I.. (2016). The effect of positive airway pressure therapy on neurocognitive functions, depression and anxiety in obesity hypoventilation syndrome. Multidiscip. Respir. Med. 11:35. doi: 10.1186/s40248-016-0071-2, PMID: 27766147 PMC5057438

[ref11] Bacon L. (2010). Health at every Size: The Surprising Truth about your Weight. Dallas, TX: BenBella Books.

[ref12] Baker K. D. Loughman A. Spencer S. J. Reichelt A. C. (2017). The impact of obesity and hypercaloric diet consumption on anxiety and emotional behavior across the lifespan. Neurosci. Biobehav. Rev. 83, 173–182. doi: 10.1016/j.neubiorev.2017.10.014, PMID: 29054731

[ref13] Balsevich G. Baumann V. Uribe A. Chen A. Schmidt M. V. (2016). Prenatal exposure to maternal obesity alters anxiety and stress coping Behaviors in aged mice. Neuroendocrinology 103, 354–368. doi: 10.1159/000439087, PMID: 26279463

[ref14] Bargain O. Zeidan J. (2019). Heterogeneous effects of obesity on mental health: evidence from Mexico. Health Econ. 28, 447–460. doi: 10.1002/hec.3852, PMID: 30739362

[ref15] Bayram N. Bilgel N. (2008). The prevalence and socio-demographic correlations of depression, anxiety and stress among a group of university students. Soc. Psychiatry Psychiatr. Epidemiol. 43, 667–672. doi: 10.1007/s00127-008-0345-x, PMID: 18398558

[ref16] Bazyk S. Winne R. (2013). A multi-tiered approach to addressing the mental health issues surrounding obesity in children and youth. Occup. Ther. Health Care 27, 84–98. doi: 10.3109/07380577.2013.785643, PMID: 23855568

[ref17] Becker E. S. Margraf J. Turke V. Soeder U. Neumer S. (2001). Obesity and mental illness in a representative sample of young women. Int. J. Obes. Relat. Metab. Disord. 25 Suppl_1, S5–S9. doi: 10.1038/sj.ijo.0801688 11466578

[ref18] Berk M. Williams L. J. Jacka F. N. O'Neil A. Pasco J. A. Moylan S.. (2013). So depression is an inflammatory disease, but where does the inflammation come from? BMC Med. 11:200. doi: 10.1186/1741-7015-11-200, PMID: 24228900 PMC3846682

[ref19] Bodenlos J. S. Lemon S. C. Schneider K. L. August M. A. Pagoto S. L. (2011). Associations of mood and anxiety disorders with obesity: comparisons by ethnicity. J. Psychosom. Res. 71, 319–324. doi: 10.1016/j.jpsychores.2011.03.004, PMID: 21999975

[ref20] Boyce W. Torsheim T. Currie C. Zambon A. (2006). The family affluence scale as a measure of national wealth: validation of an adolescent self-report measure. Soc. Indic. Res. 78, 473–487. doi: 10.1007/s11205-005-1607-6

[ref21] Bradshaw T. Mairs H. (2014). Obesity and serious mental ill health: A critical review of the literature. Healthcare 2, 166–182. doi: 10.3390/healthcare2020166, PMID: 27429268 PMC4934464

[ref22] Brislin R. W. (1970). Back-translation for cross-cultural research. J. Cross-Cult. Psychol. 1, 185–216. doi: 10.1177/135910457000100301

[ref23] Brooks R. (1996). EuroQol: The current state of play. Health Policy 37, 53–72. 10158943 10.1016/0168-8510(96)00822-6

[ref24] Brumpton B. Langhammer A. Romundstad P. Chen Y. Mai X. M. (2013). The associations of anxiety and depression symptoms with weight change and incident obesity: The HUNT study. Int. J. Obes. 37, 1268–1274. doi: 10.1038/ijo.2012.204, PMID: 23229732

[ref300] Brunwasser S. M. Gillham J. E. Eric S. K. (2009). A meta-analytic review of the Penn Resiliency Program’s effect on depressive symptoms. J. Consult. Clin. Psychol. 11, 708–715. doi: 10.1037/a0017671, PMID: 19968381 PMC4667774

[ref25] Calderon C. Forns M. Varea V. (2009). Adolescent obesity: anxiety, cognitive and behavioural symptoms characteristic of eating disorders. An. Pediatr. (Barc.) 71, 489–494. doi: 10.1016/j.anpedi.2009.07.030 19815473

[ref26] Carmichael A. R. Sue-Ling H. M. Johnston D. (2001). Quality of life after the Magenstrasse and mill procedure for morbid obesity. Obes. Surg. 11, 708–715. doi: 10.1381/09608920160558641, PMID: 11775568

[ref27] Chauvet-Gelinier J. C. Roussot A. Cottenet J. Brindisi M. C. Petit J. M. Bonin B.. (2019). Depression and obesity, data from a national administrative database study: geographic evidence for an epidemiological overlap. PLoS One 14:e0210507. doi: 10.1371/journal.pone.0210507, PMID: 30620759 PMC6324832

[ref28] Corp I. (2016). IBM SPSS Statistics for Windows, Version 24.0. Armonk, NY: IBM Corp.

[ref29] Dahm J. Wong D. N. Ponsford J. (2013). Validity of the depression anxiety stress scales in assessing depression and anxiety following traumatic brain injury. J. Affect. Disord. 151, 392–396. doi: 10.1016/j.jad.2013.06.011, PMID: 23830002

[ref30] Daumit G. L. Clark J. M. Steinwachs D. M. Graham C. M. Lehman A. Ford D. E. (2003). Prevalence and correlates of obesity in a community sample of individuals with severe and persistent mental illness. J. Nerv. Ment. Dis. 191, 799–805. doi: 10.1097/01.nmd.0000100923.20188.2d, PMID: 14671456

[ref400] Diener E. Emmons R. A. Larsen R. J. Griffin S. (1985). The Satisfaction with Life Scale. Journal of Personality Assessment. 49, 71–75. doi: 10.1207/S15327752jpa4901_13 16367493

[ref31] de Las Cuevas C. Ramallo Y. Sanz E. J. (2011). Are obesity and other physical comorbidities related to mental illness? Rev. Psiquiatr. Salud. Ment. 4, 119–125. doi: 10.1016/j.rpsm.2011.02.004, PMID: 23446192

[ref32] de Noronha S. R. Campos G. V. Abreu A. R. de Souza A. A. Chianca D. A. Jr. de Menezes R. C. (2017). High fat diet induced-obesity facilitates anxiety-like behaviors due to GABAergic impairment within the dorsomedial hypothalamus in rats. Behav. Brain Res. 316, 38–46. doi: 10.1016/j.bbr.2016.08.042, PMID: 27566182

[ref33] de Wit L. M. Fokkema M. van Straten A. Lamers F. Cuijpers P. Penninx B. W. (2010). Depressive and anxiety disorders and the association with obesity, physical, and social activities. Depress. Anxiety 27, 1057–1065. doi: 10.1002/da.20738, PMID: 20734363

[ref34] Dickerson F. B. Brown C. H. Kreyenbuhl J. A. Fang L. Goldberg R. W. Wohlheiter K.. (2006). Obesity among individuals with serious mental illness. Acta Psychiatr. Scand. 113, 306–313. doi: 10.1111/j.1600-0447.2005.00637.x, PMID: 16638075

[ref35] Esfahani S. B. Pal S. (2018). Obesity, mental health, and sexual dysfunction: A critical review. Health Psychol. Open 5:2055102918786867. doi: 10.1177/2055102918786867, PMID: 30023076 PMC6047250

[ref36] EuroQol Group (1990). EuroQol—a new facility for the measurement of health-related quality of life. Health Policy 16, 199–208. doi: 10.1016/0168-8510(90)90421-9, PMID: 10109801

[ref37] EuroQol Group (2013). EQ-5D-3L User Guide. Available at: http://www.euroqol.org (Accessed September 9, 2021).

[ref38] Fourrier C. Bosch-Bouju C. Boursereau R. Sauvant J. Aubert A. Capuron L.. (2019). Brain tumor necrosis factor-alpha mediates anxiety-like behavior in a mouse model of severe obesity. Brain Behav. Immun. 77, 25–36. doi: 10.1016/j.bbi.2018.11.316 30508579

[ref39] Frezza E. E. Shebani K. O. Wachtel M. S. (2007). Laparoscopic gastric bypass for morbid obesity decreases bodily pain, improves physical functioning, and mental and general health in women. J. Laparoendosc. Adv. Surg. Tech. A 17, 440–447. doi: 10.1089/lap.2006.0069 17705723

[ref40] Gariepy G. Nitka D. Schmitz N. (2010). The association between obesity and anxiety disorders in the population: a systematic review and meta-analysis. Int. J. Obes. 34, 407–419. doi: 10.1038/ijo.2009.252, PMID: 19997072

[ref41] Gracious B. L. Cook S. R. Meyer A. E. Chirieac M. C. Malhi N. Fischetti A. T.. (2010). Prevalence of overweight and obesity in adolescents with severe mental illness: a cross-sectional chart review. J. Clin. Psychiatry 71, 949–954. doi: 10.4088/JCP.09m05033gre 20492839

[ref200] Haddadi P. Besharat M. A. (2010). Resilience, vulnerability and mental health. Procd. Soc. Behv. 5, 639–642. doi: 10.1016/j.sbspro.2010.07.157, PMID: 30023076

[ref42] Halfon N. Larson K. Slusser W. (2013). Associations between obesity and comorbid mental health, developmental, and physical health conditions in a nationally representative sample of US children aged 10 to 17. Acad. Pediatr. 13, 6–13. doi: 10.1016/j.acap.2012.10.007, PMID: 23200634

[ref43] Hayes A. F. (2013). Methodology in the Social Sciences.Introduction to Mediation, Moderation, and Conditional Process Analysis: A Regression-Based Approach. United States: Guilford Press.

[ref44] Hayes A. F. Montoya A. K. Rockwood N. J. (2017). The analysis of mechanisms and their contingencies: PROCESS versus structural equation modeling. Australas. Mark. J. 25, 76–81. doi: 10.1016/j.ausmj.2017.02.001

[ref45] Henry J. D. Crawford J. R. (2005). The short-form version of the depression anxiety stress scales (DASS-21): construct validity and normative data in a large non-clinical sample. Br. J. Clin. Psychol. 44, 227–239. doi: 10.1348/014466505X29657 16004657

[ref46] Holt R. I. Peveler R. C. (2009). Obesity, serious mental illness and antipsychotic drugs. Diabetes Obes. Metab. 11, 665–679. doi: 10.1111/j.1463-1326.2009.01038.x, PMID: 19476478

[ref47] Iacovides A. Siamouli M. (2008). Comorbid mental and somatic disorders: an epidemiological perspective. Curr. Opin. Psychiatry 21, 417–421. doi: 10.1097/YCO.0b013e328303ba42, PMID: 18520749

[ref48] Jacobi F. Hofler M. Siegert J. Mack S. Gerschler A. Scholl L.. (2014). Twelve-month prevalence, comorbidity and correlates of mental disorders in Germany: the mental health module of the German health interview and examination survey for adults (DEGS1-MH). Int. J. Methods Psychiatr. Res. 23, 304–319. doi: 10.1002/mpr.1439, PMID: 24729411 PMC6878234

[ref49] Jarvholm K. Olbers T. Marcus C. Marild S. Gronowitz E. Friberg P.. (2012). Short-term psychological outcomes in severely obese adolescents after bariatric surgery. Obesity 20, 318–323. doi: 10.1038/oby.2011.310, PMID: 21996668

[ref50] Kehoe S. O’Hare L. (2010). The reliability and validity of the family affluence scale. Eff. Educ. 2, 155–164. doi: 10.1080/19415532.2010.524758

[ref51] Keyes C. L. M. (2005). Mental illness and/or mental health? Investigating the axioms of the complete state model of health. J. Consult. Clin. Psychol. 73, 539–548. doi: 10.1037/0022-006X.73.3.539, PMID: 15982151

[ref52] Kline P. (2000). The Handbook of Psychological Testing. 2nd *Edn*. London, New York: Routledge.

[ref53] Lovibond P. F. Lovibond S. H. (1995). The structure of negative emotional states - comparison of the depression anxiety stress scales (DASS) with the Beck depression and anxiety inventories. Behav. Res. Ther. 33, 335–343. doi: 10.1016/0005-7967(94)00075-U 7726811

[ref54] Lukat J. Becker E. S. Lavallee K. L. van der Veld W. M. Margraf J. (2016). Predictors of incidence, remission and relapse of Axis I mental disorders in young women: A transdiagnostic approach. Clin. Psychol. Psychother. 24, 322–331. doi: 10.1002/cpp.2026, PMID: 27256536

[ref55] Luppino F. S. de Wit L. M. Bouvy P. F. Stijnen T. Cuijpers P. Penninx B. W. J. H.. (2010). Overweight, obesity, and depression A systematic review and meta-analysis of longitudinal studies. Arch. Gen. Psychiatry 67, 220–229. doi: 10.1001/archgenpsychiatry.2010.2, PMID: 20194822

[ref56] Lyubomirsky S. Lepper H. S. (1999). A measure of subjective happiness: preliminary reliability and construct validation. Soc. Indic. Res. 46, 137–155. doi: 10.1023/A:1006824100041

[ref57] MacKinnon D. P. Lockwood C. M. Williams J. (2004). Confidence limits for the indirect effect: distribution of the product and resampling methods. Multivar. Behav. Res. 39, 99–128. doi: 10.1207/s15327906mbr3901_4, PMID: 20157642 PMC2821115

[ref58] Maercker A. Zhang X. C. Gao Z. H. Kochetkov Y. Lu S. Sang Z. Q.. (2015). Personal value orientations as mediated predictors of mental health: A three-culture study of Chinese, Russian, and German university students. Int. J. Clin. Health Psychol. 15, 8–17. doi: 10.1016/j.ijchp.2014.06.001, PMID: 30487817 PMC6224790

[ref59] Margraf J. Schneider S. (2017). Bochum Optimism and Mental Health (BOOM) Studies: Protocol for a Multi-National Longitudinal Research Program.

[ref600] Margraf J. Zhang X. C. Lavallee K. L. Schneider S. (2020). Longitudinal prediction of positive and negative mental health in Germany, Russia, and China. PLoS One. 15:e0234997. doi: 10.1371/journal.pone.0234997, PMID: 32574202 PMC7310683

[ref60] McIntyre R. S. Konarski J. Z. Wilkins K. Soczynska J. K. Kennedy S. H. (2006). Obesity in bipolar disorder and major depressive disorder: results from a national community health survey on mental health and well-being. Can. J. Psychiatr. 51, 274–280. doi: 10.1177/070674370605100502, PMID: 16986816

[ref61] Michael T. Zetsche U. Margraf J. (2007). Epidemiology of anxiety disorders. Psychiatry 6, 136–142. doi: 10.1016/j.mppsy.2007.01.007

[ref62] Myers A. Rosen J. C. (1999). Obesity stigmatization and coping: relation to mental health symptoms, body image, and self-esteem. Int. J. Obes. Relat. Metab. Disord. 23, 221–230. 10193866 10.1038/sj.ijo.0800765

[ref63] NCD Risk Factor Collaboration (2017). Worldwide trends in body-mass index, underweight, overweight, and obesity from 1975 to 2016: a pooled analysis of 2416 population-based measurement studies in 128.9 million children, adolescents, and adults. Lancet 390, 2627–2642. doi: 10.1016/S0140-6736(17)32129-3, PMID: 29029897 PMC5735219

[ref64] Ng F. Trauer T. Dodd S. Callaly T. Campbell S. Berk M. (2007). The validity of the 21-item version of the depression anxiety stress scales as a routine clinical outcome measure. Acta Neuropsychiatrica 19, 304–310. doi: 10.1111/j.1601-5215.2007.00217.x, PMID: 26952943

[ref65] Peduzzi P. Concato J. Kemper E. Holford T. R. Feinstein A. R. (1996). A simulation study of the number of events per variable in logistic regression analysis. J. Clin. Epidemiol. 49, 1373–1379. 8970487 10.1016/s0895-4356(96)00236-3

[ref66] Peng Z. Zheng Z. Han H. Dong C. Liang J. Lu J.. (2019). Imbalance in obesity and mental health among "little emperors" in China. PLoS One 14:e0207129. doi: 10.1371/journal.pone.0207129, PMID: 30969962 PMC6457487

[ref67] Preacher K. J. Hayes A. F. (2008). Asymptomatic and resampling strategies for assessing and comparing indirect effects in multiple mediator models. Behav. Res. Methods 40, 879–891. doi: 10.3758/BRM.40.3.879, PMID: 18697684

[ref68] Prisciandaro J. J. Roberts J. E. (2009). A comparison of the predictive abilities of dimensional and categorical models of unipolar depression in the national comorbidity survey. Psychol. Med. 39, 1087–1096. doi: 10.1017/S0033291708004522 18845012

[ref69] Roberts R. E. Duong H. T. (2016). Do anxiety disorders play a role in adolescent obesity? Ann. Behav. Med. 50, 613–621. doi: 10.1007/s12160-016-9786-8, PMID: 26961207

[ref70] Rucker D. D. Preacher K. J. Tormala Z. L. Petty R. E. (2011). Mediation analysis in social psychology: current practices and new recommendations. Soc. Personal. Psychol. Compass 5, 359–371. doi: 10.1111/j.1751-9004.2011.00355.x

[ref71] Schnohr C. W. Kreiner S. Due E. P. Currie C. Boyce W. Diderichsen F. (2008). Differential item functioning of a family affluence scale: validation study on data from HBSC 2001/02. Soc. Indic. Res. 89, 79–95. doi: 10.1007/s11205-007-9221-4

[ref72] Schumacher J. Leppert K. Gunzelmann T. Strauss B. Brahler E. (2005). The resilience scale - A questionnaire to assess resilience as a personality characteristic. Zeitschrift Fur Klinische Psychol. Psychiatrie Und Psychother. 53, 16–39.

[ref73] Scott K. M. Bruffaerts R. Simon G. E. Alonso J. Angermeyer M. de Girolamo G.. (2008). Obesity and mental disorders in the general population: results from the world mental health surveys. Int. J. Obes. 32, 192–200. doi: 10.1038/sj.ijo.0803701, PMID: 17712309 PMC2736857

[ref74] Wagnild G. M. Young H. M. (1993). Development and psychometric evaluation of the resilience scale. J. Nurs. Meas. 1, 165–178. PMID: 7850498

[ref75] Wittchen H. U. Hofler M. Gloster A. T. Craske M. G. Beesdo K. (2011). “Options and Dilemmas of Dimensional Measures for DSM-5: Which Types of Measures Fare Best in Predicting Course and Outcome?,” in The Conceptual Evolution of DSM-5. Washington, 119–143.

[ref100] WHO (2000). Cross-national comparisons of the prevalences and correlates of mental disorders. WHO International Consortium in Psychiatric Epidemiology. Bulletin of the World Health Organization 78, 413–426. PMID: 10885160 PMC2560724

